# Supplementary material for: In Vitro Effects on Thrombin of Paris Saponins and In Vivo Hemostatic Activity Evaluation of Paris fargesii var. brevipetala
Source: Molecules. 2019 Apr 11;24(7):1420. doi: 10.3390/molecules24071420 (PMC6480468; doi:10.3390/molecules24071420)
Supplement: Supplementary file 1 [file molecules-24-01420-s001.pdf]

Table S1: Data of the coagulation time in vitro of thrombin (n=5)

M: thrombin + ultrapure water; M+H: thrombin + PS H; M+Q: thrombin + PF saponins extract; coagulation did not occur in inactivated thrombin + PS H group;

Groups	M	M+H	M+Q
Time(s)	41.02±0.90	35.24±0.69	22.01±0.37

Table S2: Data of bleeding time (BT) in mice and Hemostasis effect of PS H and PF saponins extract (n=8)

Hemostatic effect =100% × (blank group BT -drug group BT)/blank group BT

C：control(0.2% CMC-Na solution)；H: PS H(H32, 32 mg/kg; H20, 20 mg/kg; H8, 8 mg/kg)；Q: PF saponins extract (Q32, 32 mg/kg; Q16, 16 mg/kg)； B: Yunnan Baiyao (1 g/kg) ;* compared with 0.2% CMC-Na ($p<0.05$)

Groups	C	H32	H20	H8	Q32	Q16	B
BT (s)	11.53±0.24	5.61±0.75*	5.79±1.05*	7.36±0.86*	5.05±0.70*	8.81±0.79*	7.53±1.39*
Hemostatic effect (%)		51.34	49.78	36.17	56.2	23.59	34.69

Table S3: Data of the 4 blood coagulation parameters of PS H and PF saponins extract in rats (n=8)

C：control(0.2% CMC-Na solution)；H: PS H(H20, 20 mg/kg; H12.5, 12.5 mg/kg; H5, 5 mg/kg)；Q: PF saponins extract (Q20, 20 mg/kg; Q10, 10mg/kg)；G: Gongxuenin (80 mg/kg)；B: Yunnan Baiyao (0.6 g/kg); * compared with 0.2% CMC-Na ($p<0.05$); Prothrombin time (PT); Thrombin time (TT); Activated partial thromboplatin time (APTT); Fibrinogen (FIB);

Groups	C	H20	H12.5	H5	Q20	Q10	G	B
PT (s)	9.91±0.31	10.95±0.18*	9.01±0.14*	10.69±0.31	9.38±0.11	9.09±0.96*	10.13±0.35	10.18±0.33
APTT(s)	17.16±1.75	15.25±0.42	17.35±2.35	15.64±0.66	14.68±0.35	14.96±0.37	14.73±0.21	14.38±0.29
TT (s)	56.08±3.36	59.13±0.90	55.46±4.70	56.25±1.78	54.63±0.95	55.89±0.80	56.14±1.16	55.65±1.12
FIB (g/L)	1.11±0.21	2.05±0.15*	1.88±0.16*	1.63±0.09*	2.46±0.20*	1.77±0.12*	1.97±0.09*	1.99±0.14*

Table S4: Data of the liver function and blood lipid parameters of PS H and PF saponins extract in rats (n=8). C：control(0.2% CMC-Na solution)；H: PS H(H20, 20

mg/kg; H12.5, 12.5 mg/kg; H5, 5 mg/kg)；Q: PF saponins extract；G: Gongxuenin (80 mg/kg)；B: Yunnan Baiyao (0.6 g/kg); * compared with 0.2% CMC-Na ($p<0.05$); Alanine aminotransferase (ALT), Aspartate aminotransferase (AST), Albumin (ALB), Total cholesterol (CHOL), Triglyceride (TG), total proteins (TP), High density lipoprotein (HDL) and Low density lipoprotein (LDL)

Groups	C	H20	H12.5	H5	Q20	Q10	G	B
ALT U/L	16.50±1.71	25.67±3.70	27.20±1.85*	26.67±3.14	30.25±3.22*	31.18±2.26*	25.67±4.80	24.80±2.06
AST U/L	74.00±3.49	104.00±9.63*	78.86±3.54	104.36±9.28*	111.00±17.13*	96.88±10.18	83.71±5.48	81.25±7.88
TP g/L	51.23±2.10	56.23±2.70	52.54±1.63	57.40±1.85	50.40±3.45	56.33±1.92	52.26±2.29	58.68±5.64
ALB g/L	28.89±1.15	31.73±1.13	30.35±0.58	32.77±0.92	29.50±1.85	30.61±0.7	29.14±0.75	32.48±1.84
CHOL m mol/L	1.57±0.12	1.72±0.09	1.86±0.14	1.68±0.11	1.51±0.16	1.40±0.08	1.92±0.13	1.80±0.18
TG m mol/L	0.09±0.02	0.25±0.06	0.09±0.01	0.28±0.09	0.18±0.04	0.12±0.02	0.13±0.02	0.16±0.05
HDL m mol/L	0.28±0.02	0.29±0.01	0.35±0.02	0.29±0.01	0.28±0.04	0.26±0.02	0.35±0.03	0.33±0.03
LDL m mol/L	0.10±0.01	0.14±0.02	0.12±0.01	0.11±0.01	0.11±0.01	0.09±0.01	0.11±0.01	0.12±0.02
